# Macrophages in cardiac repair: Environmental cues and therapeutic strategies

**DOI:** 10.1038/s12276-019-0269-4

**Published:** 2019-12-19

**Authors:** Geoffrey de Couto

**Affiliations:** Smidt Heart Institute, Cedars-Sinai Medical Center, 8700 Beverly Blvd., Los Angeles, CA 90048 USA

**Keywords:** Cardiovascular diseases, Heart stem cells

## Abstract

Mammals, in contrast to urodeles and teleost fish, lose the ability to regenerate their hearts soon after birth. Central to this regenerative response are cardiac macrophages, which comprise a heterogeneous population of cells with origins from the yolk sac, fetal liver, and bone marrow. These cardiac macrophages maintain residency in the myocardium through local proliferation and partial replacement over time by circulating monocytes. The intrinsic plasticity of cardiac macrophages in the adult heart promotes dynamic phenotypic changes in response to environmental cues, which may either protect against injury or promote maladaptive remodeling. Thus, therapeutic strategies promoting myocardial repair are warranted. Adult stromal cell-derived exosomes have shown therapeutic promise by skewing macrophages toward a cardioprotective phenotype. While several key exosomal non-coding RNA have been identified, additional factors responsible for cardiomyocyte proliferation remain to be elucidated. Here I review cardiac macrophages in development and following injury, unravel environmental cues modulating macrophage activation, and assess novel approaches for targeted delivery.

## Heart disease: Rebooting immune cells to repair the heart

The human heart may be coaxed toward regeneration by modifying the activity of specialized immune cells known as macrophages. Insight from the regenerating hearts of zebrafish, newt, and neonatal mammals has revealed that macrophages are required to replace scar with functioning heart tissue. As mammals lose the ability to regenerate heart tissue, macrophages mature from a regenerative phenotype towards an immunomodulatory phenotype. By adulthood, heart macrophages comprise a mixed population of cells arising from either early embryonic development or differentiation from white blood cells. In this issue, Dr. Geoffrey de Couto from the Smidt Heart Institute at Cedars-Sinai Medical Center, reviews the role of macrophages in heart repair and therapeutic strategies to enhance their activity. Recent studies suggest that exosomes, which are naturally-released nano-sized vesicles, can re-educate adult macrophages to protect the heart from injury.

## Introduction

The mammalian heart develops within the womb, obtaining independent function from its host, and maintaining continuous contractile activity until death. In contrast to other organs, the developed heart has minimal renewal over the course of life (~0.5–2%/year) and limited regenerative capacity following injury^1^. Cardiomyocytes occupy the greatest proportion of space (70–85%, by volume), but contribute only 30–40% of the total number of cells within the heart. The remaining cell types include fibroblasts, endothelial cells, perivascular cells, and macrophages^2^. Several studies, including those investigating amphibians^3,4^, have revealed the importance of resident and non-resident macrophages to the development and homeostasis of the mammalian heart^5–11^. In this review, I focus on cardiac macrophages during development and following injury, environmental cues modulating macrophage phenotype, exosomes as therapeutic entities, and bioengineering approaches for enhanced delivery.

## Origins: resident versus non-resident

Macrophages are mononuclear phagocytes and essential components of the innate immune system (i.e., monocytes, neutrophils, basophils, eosinophils, mast cells, dendritic cells, natural killer cells). In 1968, van Furth and Cohn revealed that macrophages are primarily derived from blood monocytes^12^; a classification now commonly referred to as non-resident or monocyte-derived macrophages. Monocytes differentiate from hematopoietic stem cells in the bone marrow and splenic reservoir with the presence of several cytokines including M-CSF, GM-CSF, IL-1β, and IL-3^13,14^. Once mobilized into the peripheral circulation, these cells are classified into two main populations coexisting at steady-state^15,16^: (1) patrolling monocytes (Ly-6C^lo^ in mice, CD14^lo^CD16^+^ in humans), which survey the vascular lumen, scavenge oxidized lipids, and clear cellular debris; and (2) inflammatory monocytes (Ly-6C^hi^ in mice, CD14^hi^CD16^−^ in humans), which secrete an abundance of proinflammatory cytokines. Based on these findings, the long-held perspective was monocytes populate all forms of macrophages throughout the body, including those found residing in tissue. It was not until recently, with the advent of fate mapping experiments, that two distinct populations of resident macrophages (microglia and Langerhans cells), were traced back to the prenatal yolk sac and fetal liver, respectively^17,18^. As a result of these findings, the origins of other tissue resident macrophages have been questioned. Now it is clear most tissue resident macrophages, including those found in the liver, spleen, lung, peritoneum, and heart, originate from the yolk sac^5,19,20^; one notable exception are intestinal resident CX3CR1^+^ macrophages, which are seeded exclusively by circulating Ly6-C^hi^CCR2^+^ monocytes^21^. In contrast to monocytes, yolk sac-derived resident tissue macrophages express CX3CR1^hi^CCR2^-^MHC-II^lo5,6^, differentiate independently of Myb^18^, and persist into adulthood under steady-state conditions through local proliferation^20^, but may be partially replenished by blood monocytes over time^5,6^ (described in greater detail below).

## Nodal regulators of inflammation

Macrophages modulate the inflammatory environment during homeostasis or injury through direct and indirect interactions with the surrounding milieu. In the infarcted myocardium, macrophages attenuate injury by secreting cytoprotective factors (e.g., IL-10^22,23^, myeloid-derived growth factor^24^, fibroblast growth factor-1^25^) and scavenging cell debris (i.e., efferocytosis^26,27^). The compounding effects from these changes dampen inflammatory cell infiltrates, reduce proinflammatory cytokine/chemokine/growth factor release (e.g., TNFα, IL-1β, MCP-1, TGFβ), and suppress myofibroblast activation and fibrosis^28^. As professional antigen-presenting cells, macrophages bridge the innate and adaptive immune systems by processing foreign antigens into peptides and presenting them to T cells with major-histocompatibility complex class I and class II molecules. This cross-talk is of particular importance in chronic diseases as adaptive immunity is a primary effector sustaining disease pathology^29^. In a model of ischemia-induced heart failure, adoptive transfer of splenic CD4 + T cells from heart failure mice into naive control mice promotes adverse cardiac remodeling with left ventricular systolic dysfunction and hypertrophy^30^. Furthermore, in a model of pressure overload-induced heart failure, early suppression of CCR2 + monocyte-derived macrophages attenuate lymph node CD3 + T cell expansion and cardiac hypertrophy^31^. Collectively, these data demonstrate the centralized role of macrophages in modulating the inflammatory response within the heart.

## Regenerative response

The mammalian adult heart is incapable of regenerating itself following injury. Instead, cardiomyocyte loss resulting from myocardial infarction (MI) replaces functional contractile myocardium with a non-contractile scar. However, this non-cardioregenerative process is inconsistent across species. In adult teleost fish (e.g., zebrafish) and urodele amphibians (e.g., newts), apical resection leads to complete regeneration of the myocardium without scar formation^32–35^, suggesting that their myocardial regenerative process is maintained throughout life; n.b., medaka teleost fish are unable to regenerate their hearts and promote scar formation like adult mammalian hearts^36^. The regenerative process begins with clot formation, inflammatory cell infiltration, as well as fibrin and extracellular matrix (ECM) deposition^33,34,37^; structural proteins are integral to the initial healing process to prevent blood loss and maintain the structural integrity of the contractile organ. During the following weeks, cardiomyocyte proliferation ensues through cardiomyocyte de-differentiation followed by cell-cycle re-entry with a gene expression pattern reflective of fetal development (e.g., *Gata4*, *Isl1*)^33,34^. Within 60–90 days, functional cardiomyocytes have replaced the ECM to produce hearts appearing structurally and functionally normal^33,34^.

Neonatal, but not adult, mammalian hearts retain the ability to regenerate following injury for a limited time after birth^38–40^. Like zebrafish and newts, 1-day old neonatal mice with myocardial apical resection or MI regenerate the injured myocardium through clot formation, inflammatory cell infiltration, and cardiomyocyte de-differentiation with activation of the cardiomyocyte fetal gene programs^38,39^. However, if the same protocol is performed on > 7-day old neonatal mice, the regenerative response is lost and the prototypical adult mammalian scar results^38,39^. Similar results have been observed in larger mammals. Neonatal pigs surgically induced to receive MI on postnatal days 1, 2, or 3, regenerate their hearts within 2 weeks of injury with preserved myocardial function and architecture, minimal scar formation, and increased cardiomyocyte cell-cycle activity^41^. Validating neonatal myocardial regeneration in humans has been challenging due to limited sample numbers and sizes. Case reports dating back to 1966 demonstrate that newborn patients surviving MI soon after birth regain structural and functional capacity within weeks of injury^42–44^. More recently, cardiomyocytes isolated from healthy neonate patients ( < 6 months) were shown to contain greater proportions of proliferating cardiomyocytes relative to age-matched patients with heart disease (<6 months) or healthy adult patients (up to 30 years of age)^43,45^. Together, these data suggest that mammalian cardiac regeneration occurs within the first few days of birth but is rapidly lost with age.

In all mechanistic studies detailing postnatal cardiac regeneration (i.e., small animal studies), a robust inflammatory cell infiltrate is observed soon after injury^3,7,37^. Although this response mirrors the infiltrate observed in non-regenerating hearts (i.e., rapid neutrophil influx followed by macrophage infiltration), regenerating hearts retain macrophages for a longer period following injury^7,37^. In teleost cryoinjured hearts, where zebrafish but not medaka elicit a regenerative response, zebrafish have an elevated and sustained macrophage presence following injury, whereas neutrophil infiltrate is similar between both fish^37^. This observation parallels the findings in regenerating neonatal mouse hearts. Myocardial infarction introduced to mice on postnatal day 1 (P1; regenerative) relative to P7 or P14 (non-regenerative) have sustained macrophage infiltration in the heart 7 days following injury^7^. When macrophages are selectively depleted with clodronate liposomes during the early-phase of repair in zebrafish, newts, or neonatal mice, the cardiac regenerative response is lost^3,7,37^. It is not fully understood *how* macrophages coordinate the cardiac regenerative response, but several pathways activated during regeneration are associated with known macrophage function: axonal regrowth^46,47^, angiogenesis^7,48^, ECM degradation^49^, and efferocytosis^26,50^. Together, these data highlight the central importance of macrophages in myocardial regeneration across species, which parallels the regenerative response observed in other tissues and organs.

## Non-regenerative response: repair of the adult mammalian heart following MI

The mammalian heart loses its regenerative abilities soon after birth. During this narrow timeframe, ventricular resection or MI leads to reconstruction of the myocardial architecture to the point it is nearly indistinguishable both morphologically and functionally from non-infarcted tissue, bar some residual fibrosis^38,39^. However, when MI is performed at P7 or later, the regenerative process is lost^39^. In this setting, extensive cardiomyocyte death precedes a sequence of three characteristic events of scar formation^51^. In phase I (the inflammatory phase), an intense but transient, influx of neutrophils and macrophages swarm the infarct region to resolve the harsh inflammatory environment^52^. Inundated by an array of endogenous alarmins (e.g., high mobility group B1, heat shock proteins) and proinflammatory cytokines, the innate immune infiltrate blunts collateral damage through extensive clearance of cellular and ECM debris^53^. In phase II (the proliferative phase), when the intense inflammatory phase has subsided, macrophages secrete chemokines to recruit and activate fibroblasts and endothelial cells. One of the prominent factors released is transforming growth factor-β (TGF-β), which simulates the conversion of fibroblasts into myofibroblasts and, in turn, the vast production and deposition of ECM proteins for scar formation^54,55^. In phase III (the maturation phase), following apoptosis of the reparative macrophages in phase II, the infarct evolves into a mature scar with cross-linked collagen fibers^56,57^.

**Fig. 1:**
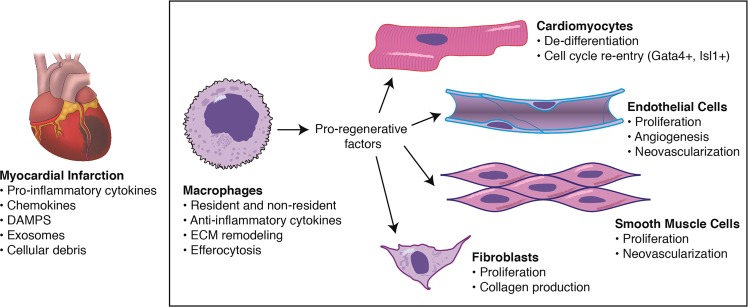
Macrophages orchestrate the regenerative process post-MI. Resident and non-resident macrophages respond to environmental cues released from the ischemic myocardium and secrete pro-regenerative factors to cardiac cell populations. *DAMPS* danger-associated molecular patterns, *ECM* extracellular matrix

Macrophages are essential for remodeling the adult mammalian heart post-MI (Fig. 1). Selective depletion of macrophages following cryoinjury or MI results in severely compromised myocardial architecture, which reveals unresolved cellular debris and heightened collagen deposition, and increased mortality^10,11^. To better understand why a reparative disparity exists between young and old hearts, it is important to assess the physiological processes that align with the dramatic temporal shift during development from robust to minimal regenerative ability of the heart. The loss of cardiomyocyte proliferative capacity has been linked to dramatic changes in the oxygen levels between the fetal circulation and the first few days of life^58^. Soon after birth the oxygen tension increases from a PO2 of 32–35 mm Hg (fetal) to a PO2 of 25–28 mm Hg (postpartum) and correlates with an increase in mitochondrial content and complexity. The subsequent shift from a glycolytic to oxidative metabolism induces reactive oxygen species (ROS) production promoting cardiomyocyte cell cycle arrest through the DNA damage response^58^. In parallel with blood oxygenation changes is a shift in immune cell function. Macrophages are required for both regenerative and non-regenerative responses, but the discrepancy in outcomes between neonatal and adult hearts following injury suggests alteration of their function after birth. In amphibians, loss of regenerative ability (anurans) following metamorphosis coincides with maturation of the immune system, while preservation of regenerative ability (urodeles) parallels a more conservative adjustment to immune development^4^. The shift in macrophage population is consistent with recent findings in mice reflecting dynamic changes in macrophage residency with age and disease^5,59^. Single-cell transcriptomic data reveal at least four populations of resident cardiac macrophages exist in the adult heart^6^, including resident macrophages maintained through local proliferation (CCR2^−^TIMD4^+^LYVE1^+^MHC-II^lo^), resident macrophages partially replaced by monocytes (CCR2^−^TIMD4^−^LYVE1^−^MHC-II^hi^), and two CCR2^+^MHC-II^hi^ populations fully replaced by monocytes. Lineage tracing studies of resident macrophages (CX3CR1^+^CCR2^−^) revealed distinct repopulation dynamics following MI: CCR2^−^TIMD4^+^LYVE1^+^MHC-II^lo^ decreased to ~83%, while CCR2^−^TIMD4^−^LYVE1^−^MHC-II^hi^ decreased to ~7%, of their original populations at steady state. Selective depletion of CX3CR1^+^ macrophages prior to MI impaired infarct healing, reduced cardiac function, and increased mortality^6^. Several studies to date demonstrate that macrophages are required for efficient cardiac repair in the neonate^7,60^ and adult^10,11,60^ heart. Despite long-term residence of CX3CR1^+^CCR2^−^ macrophages from birth until adulthood^5,6^, it is unclear if any *adult* cardiac macrophage population, whether yolk sac- or monocyte-derived, supports a regenerative response post-MI. Therapeutic manipulation of distinct resident and/or non-resident adult macrophage populations may prove to be a more powerful tool for enhancing repair.

## Environmental cues and activation states: a role for exosomes

Macrophage activation is required to appropriately respond to dynamic shifts in environmental cues. In the post-infarct myocardium, two sequential sets of monocytes infiltrate the myocardium from the bone marrow and splenic reserve^14,16^. In the first wave, proinflammatory monocytes (CCR2^+^Ly-6C^hi^) are recruited (e.g., CCL2 and CCL7) soon after injury. These monocytes differentiate into proinflammatory macrophages that secrete proteolytic enzymes and promote efferocytosis. In the second wave, a small set of monocytes (CCR2^+^Ly-6C^lo^) are recruited to the site of injury and differentiate into Ly-6C^lo^ macrophages to facilitate wound repair through myofibroblast activation, angiogenesis, and extracellular matrix deposition; previously recruited Ly-6C^hi^ monocytes convert to Ly-6C^lo^ macrophages and contribute to wound repair^52^. The most prominent, but perhaps dated, nomenclature for macrophage activation is M1 (classical activation; stimulated by LPS and/or IFNg) and M2 (alternative activation; stimulated via IL-4)^61^. These dichotomous terms were used to reflect the first (M1) and second (M2) waves of macrophage infiltrates into damaged tissue but were later found to more clearly reflect two ends of a spectrum of activation states. This concept spurred the identification and classification of numerous additional subsets, such as M2a, M2b, and M2c, each representing activation based on distinct factors^62^. To simplify the rapidly evolving nomenclature, a common framework for macrophage-activation was proposed to clearly identify the protein factor(s) used to promote activation (e.g., IL-4-stimulated macrophages denoted as M(IL-4))^63^. Despite a better understanding of environmental cues, adult macrophages have yet to replicate the cardioregenerative abilities observed in neonates.

### Extracellular vesicles

**Fig. 2:**
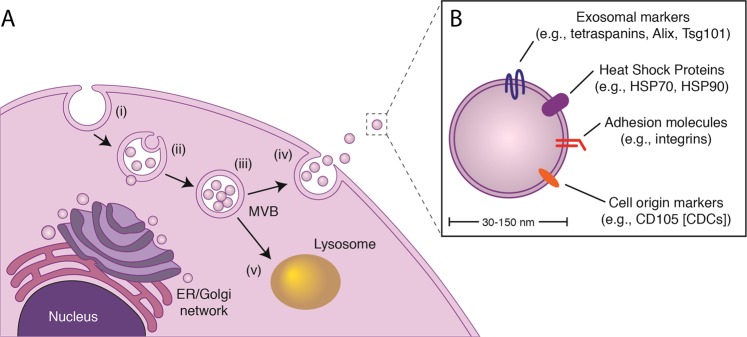
Exosome biogenesis and release. **a**, Exosome production follows a stepwise sequence of events. (i), inward invagination of the plasma membrane to form an endosome; (ii), loading of exosomal cargo into the endosome, which leads to the formation of a multivisicular body (MVB); (iii), transport of the MVB to the membrane; and (iv), fusion of the MVB to the plasma membrane releasing exosomes. (v), MVBs not destined for extracellular release fuse with lysosomes for degradation. **b**, Exosomes contain an abundance of cargo reflective of the cell of origin. ER *Endoplasmic* *reticulum*

Cells secrete extracellular vesicles (EVs) in both health and disease^64^. Over the past decade, detailed examination of EV biogenesis has led to the classification of EVs into at least two distinct groups: (1) exosomes (30–150 nm), which develop within endosomes as multivesicular bodies; and (2) microvesicles (150–500 nm), which arise from plasma membrane budding. Exosomes, which were originally classified as cellular waste, have garnered attention within the scientific community following the discovery of proteins, RNAs (predominantly non-coding RNAs; ncRNA), and lipids within their cargo. Although DNA fragments have been associated with some exosomes, their role remains to be clarified^65,66^. Exosome biogenesis involves a 4-step process (Fig. 2): (i) endosome formation, (ii) inward invagination of the endosomal membrane to form multivesicular bodies (MVBs; exosomal membranes within the MVB are enriched in cholesterol, sphingomyelin, phosphatidyl serine, and ceramide), (iii) transport of the MVBs to the plasma membrane, and (iv) fusion of the MVB with the plasma membrane for exosomal release into the extracellular space. While the molecular mechanisms governing this process remain to be fully elucidated, two processes contribute: the endosomal sorting complex required for transport (ESCRT)-dependent pathway^67,68^, and the ESCRT-independent pathway of lipid (e.g., ceramide) synthesis^69^. As a result, several proteins involved in these processes are found on the surface of exosomes (e.g., Flotillin, Alix, Tsg101, and tetraspanins, such as CD9, CD63, and CD81). Internally, exosomes contain bioactive cargo protected from the extracellular space by the lipid bilayer membrane, rendering the contents resistant to protease and RNase digestion^70,71^. Proteomic and RNA-sequencing analyses have unveiled a rich catalog of exosomal components; some are consistent across exosome sources, but most vary greatly, reflecting the cell of origin and the culture conditions (e.g., oxygen tension^72^). Data to date support the hypothesis that most of the functional effects of exosomes are attributable to their ncRNA cargo: light permeabilization of exosomes in the presence of RNase undermines bioactivity^73^, and most of the RNA content of exosomes is noncoding^74^. The resulting molecular signatures have garnered attention for their utility as clinical biomarkers (e.g., cancer) and therapeutic entities.

### Exosome secretion post-MI

The heart is comprised of multiple cell types (i.e., cardiomyocytes, endothelial cells, fibroblasts, smooth muscle cells, and macrophages) working in unison to generate a functional, beating myocardium. All cardiac cells and are known to secrete an array of cytokines and chemokines in response to stress, but more recent work using electron microscopy and immunocytochemistry reveal exosomes as an essential component of the myocardial paracrine network^75^. Exosomes produced from each cell type are reflective of the cell stress. Cardiomyocytes, for example, secrete exosomes with proangiogenic miR-222 and miR-143 in response to ischemic stress^76^, but may also package functional glucose transport proteins (e.g., GLUT1 and GLUT4) in response to glucose deprivation^77^. Exosomes derived from endothelial cells exposed to various stressors can promote angiogenesis (i.e., miR-214)^78^, suppress smooth muscle cell proliferation (i.e., miR-143/145)^79^, and enhance inflammatory cell attachment (i.e., ICAM-1 and TNFAIP3)^80^. Other disease-specific stimuli, such as 16kDa N-terminal prolactin fragment driving peripartum cardiomyopathy, promote exosomal transfer miR-146a to cardiomyocytes where it suppresses metabolic function^81^. Although endothelial cell exosomes suppress smooth muscle cell proliferation, smooth muscle cell-derived exosomes may reciprocally enhance endothelial cell migratory and angiogenic effects via exosomal transfer of miR-143^82^. Cardiac fibroblasts, which comprise ~15% of the cell population within the myocardium^2^, have robust regulatory effects on cardiomyocytes. When exposed to angiotensin II, cardiac fibroblasts secrete exosomes containing miR-21 and miR-423 which promote cardiomyocyte hypertrophy^83^ through activation of the MAP/Akt signaling pathway^84^. Lastly, cardiac macrophages utilize exosomes to transfer miR-155 to fibroblasts in order to suppress fibroblast proliferation, decrease collagen production, and promote cardiac rupture^85^. Together, these data highlight the intercellular communication pathways between cardiac cell types via exosomes and elucidate some of the complex environmental cues within the ischemic myocardium.

## Therapeutic targeting

**Fig. 3:**
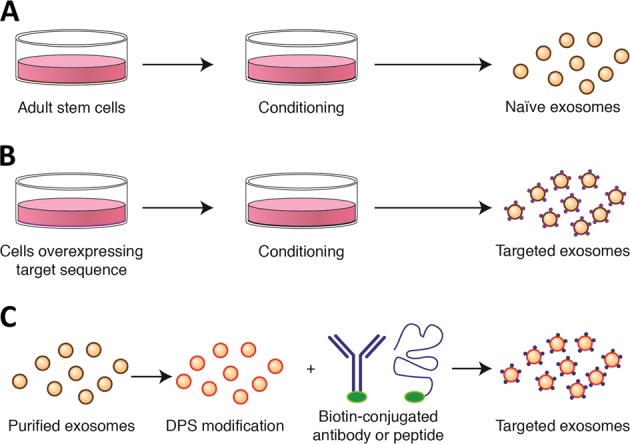
Bioengineering exosomes for targeted therapeutics. **a** Exosomes from unmodified (naïve) adult stem cells can be collected in vitro following a period of conditioning. **b** Cells can be modified to overexpress and incorporate distinct targeting proteins into the exosomal membrane. **c** Naïve exosomes may be collected and modified after purification for targeted delivery. DPS: DMPE-PEG-streptavidin

In addition to the complexities of exosome communication within the host myocardium, exogenous cell-derived exosomes may provide therapeutic utility for repair (Fig. 3). Many insights into exosome therapeutics have resulted from work within the field of regenerative medicine.

### Adult stromal cell exosomes for cardioprotection

Cell therapy for MI has been tested for well over a decade in both small and large animal models with varying degrees of success. Despite limited retention following intramyocardial or intracoronary delivery, distinct adult stromal cell populations (e.g., mesenchymal stem cells [MSCs], cardiosphere-derived cells [CDCs]) confer long-lasting protection to the host myocardium, which include enhanced angiogenesis, reduced scar formation, and improved cardiac function^9,86–88^. Bone marrow-derived MSCs (BM-MSCs) have been described to modify macrophage phenotype toward an M2-like status, with reductions in TNFa, IL-6, IL-1b, IL-12, iNOS, and CD86, but increased expression of IL-10, IL-4, CD206, and Arg1^8^. In a mouse model of MI, intramyocardial delivery of BM-MSCs increased F4/80 + CD206 + macrophages in the heart, improved cardiac function, and prevented mortality^8^. While the mechanism of macrophage activation was not explored, recent work using CDCs (i.e., adult stromal cells derived from cardiac tissue^89^) reveal that adult stromal cell-derived exosomes may be isolated in vitro (Fig. 3a) and, when delivered in vivo, recapitulate the cardioprotective effects of cell therapy^9,87,90,91^. When delivered acutely post-MI, CDCs and their secreted exosomes polarize macrophages (M_CDC_ and M_CDCexo_, respectively) to a cardioprotective state suppressing the proinflammatory cytokine expression and promoting efferocytosis^90^. RNA-sequencing of both exosomes and M_CDCexo_ revealed that exosomal transfer of miR-181b to macrophages is responsible, at least in part, for the cardioprotective response post-MI^90^. While miRs are important for exosome bioactivity^74,92^, exosomes comprise a much larger fraction of other non-coding RNA (ncRNA)^92^, such as long ncRNA (lncRNA), Y RNA, and snoRNA. Recently the most abundant RNA in CDC-derived exosomes, a Y RNA fragment dubbed EV-YF1, was found to be packaged and transferred to macrophages^22^, which increased IL-10 expression and protection against ischemic insult^22^. Each of these studies provide insight into mechanisms that require targeting for enhanced cardiac repair. It is likely a composite therapeutic strategy targeting multiple cell types, rather than a single molecular entity focused on single cell population, will be required to promote a cardioregenerative response in adults.

### Engineered exosomes for delivery to the heart

Exosomes contain a variety of surface proteins reflecting identity, cell of origin, and binding/uptake. One of the main challenges in the field of exosome therapeutics is improving delivery to the cell/tissue of interest. Although direct intramyocardial injections are feasible, the translational application into humans is highly invasive and technically challenging. Therefore, a less invasive route of administration, such as intravenous delivery, is preferable. Under steady state conditions, exosomes delivered intravenously accumulate in the liver, lungs, and spleen^93,94^, albeit at different proportions depending on cell source^94^, and are readily taken up by monocytes/macrophages^93^. Following MI, exosomes are enriched within the infarct area and may reflect uptake by local macrophages or other immune cells^90^. Despite enrichment at the site of injury and a natural avidity for uptake by macrophages, relatively low exosome concentrations within the ischemic area may not be sufficient to confer therapeutic benefit. To reduce off-target delivery, strategies enhancing delivery of exosomes to defined cells is of interest to the field.

Several studies to date have shown promising results for targeted delivery to the heart. One approach is to engineer exosomes with a specific protein through a producer cell (Fig. 3b). For example, in order to produce exosomes containing angiotensin type II receptor 1 (ATR1) on its surface, HEK293 cells were used to overexpress ATR1 and then were exposed to hypotonic conditioning (143 mOsm/kg; osmotic stretch) or angiotensin II stimulation^95^. The resulting conditioned media was collected, and the exosomes purified. To assess whether ATR1 exosomes were functional in vivo, AT1R exosomes or PBS were delivered intravenously into AT1R knockout mice. Twenty-four hours later, AT1R knockout mice treated with AT1R exosomes, in contrast to PBS, responded to angiotensin II infusion with a 30% increase in systolic pressure^95^. A modification to the overexpression approach is a technology known as ‘surface display’^96^, whereby a specific protein sequence is fused to the C1C12 domain of the lactadherin domain for display on the exosomal membrane surface. To localize exosomes to the ischemic myocardium, an ischemic peptide was fused to the C1C12 domain and overexpressed in HEK293 cells^97^. The resulting exosomes isolated from in vitro conditioned media revealed enhanced localization to necrotic cardiomyocytes in vitro and the ischemic myocardium in vivo^97^. A more versatile approach to exosomal targeting is a technology known as ‘cloaking’ (Fig. 3c), which is a modification that may be applied directly to the exosome rather than the producer cell^97^. To do so, modified glycerol-phospholipid-PEG (DMPE-PEG) is conjugated with streptavidin (DMPE-PEG-Streptavidin; DPS) to generate an anchor embedded directly into the exosomal membrane so any biotinylated molecule, either antibody or protein, may be non-covalently linked to the exosome. The utility of this technology was demonstrated in vivo after exosomes were cloaked with or without a biotin-tagged ischemic peptide and then delivered intravenously after MI. Exosomes cloaked with the biotin-tagged ischemic peptide showed a 3-fold enrichment within the infarct region of the heart^97^. Together, these technologies reveal broad steps in the field of exosome bioengineering for targeted therapeutics. Despite being in its infancy, the field has made significant strides forward and will undoubtedly provoke additional technologies in the near future.

## Conclusions and perspectives

Cardiac macrophages play a fundamental role in the mammalian heart and contribute to both the regenerative (neonatal) and non-regenerative (adult) response post-MI. It is now clear there are two dominant pathways of macrophage development in mammals. The first relies on sequential steps of differentiation from hematopoietic stem cell, monocytes, and then macrophages. Under homeostatic conditions, monocytes reside in bone marrow and splenic reserves, but are mobilized into the peripheral circulation to maintain homeostasis, repopulate distinct resident macrophage populations, and respond to injury. The second involves early embryonic seeding into developing tissues. Resident tissue macrophages populate the heart from the yolk sac and fetal liver but are gradually replaced by invading monocyte populations resulting in a mixed population of macrophages in adulthood. Whether a specific subset of resident cardiac macrophages, which are depleted following MI, are required for enhanced regeneration remains to be answered. Data to date suggests that maturation, which may reflect changes in local environmental cues (i.e., protein factors and exosomes) and modifications of the epigenome, limits the regenerative ability of macrophages. However, additional experimental studies are required to better understand *how* macrophage maturation contributes to regeneration and disease progression.

Adult stromal cell-derived exosomes are effective therapeutic agents for cardioprotection post-MI. To date, several important exosomal ncRNA and protein cargos responsible for stimulating macrophage-dependent cardioprotection have been identified, but many remain undiscovered. Although studies within this review focus on mammalian exosomes, important clues may lie within exosomal ncRNA from highly regenerative amphibians like the newt. Recent studies have demonstrated newt A1 myogenic precursor cell EVs contain a greater abundance of both protein and RNA per particle relative to mammalian counterparts^98^. Despite millennia of evolutionary divergence, newt exosomes retain conserved RNA and protein cargo that protect mammalian cardiomyocytes against oxidative stress.

**Fig. 4:**
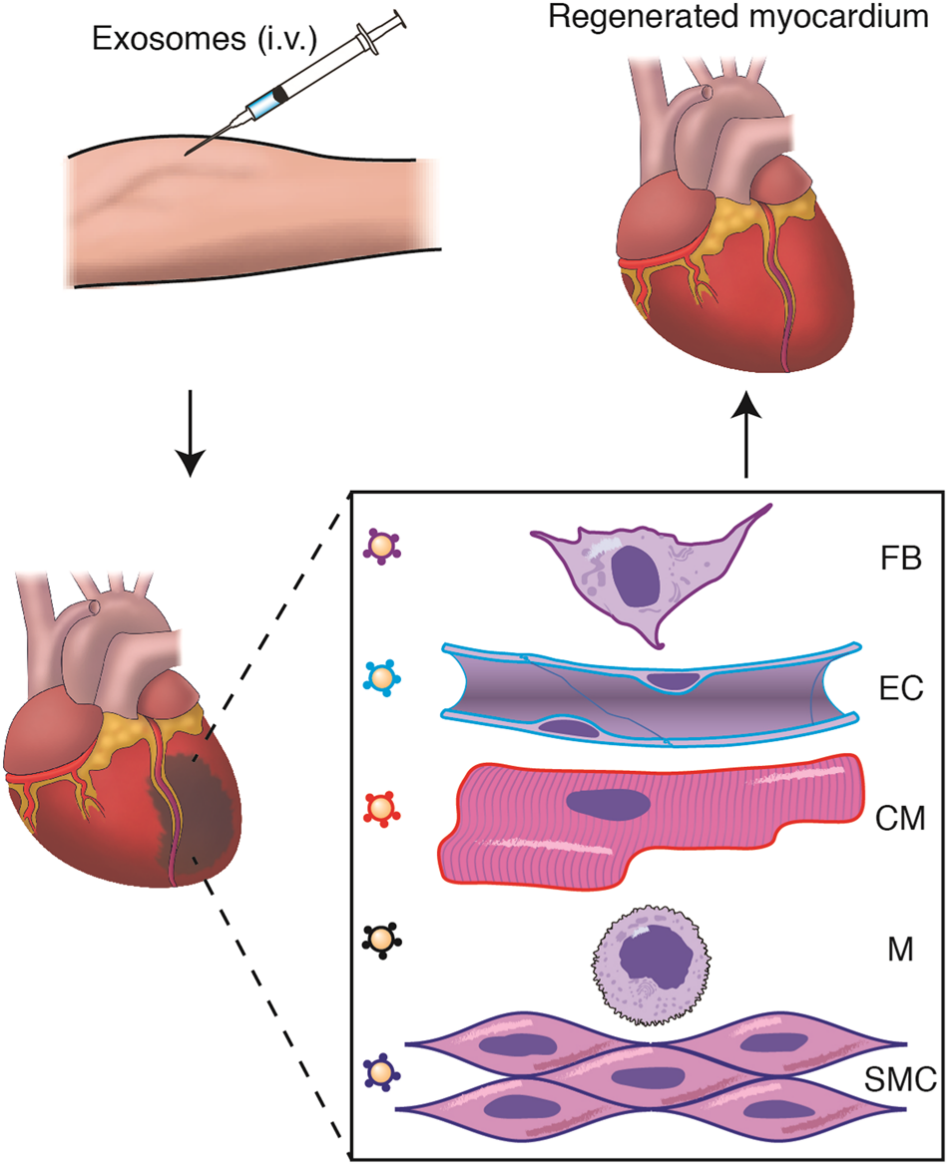
Multipronged therapeutic approach for MI. Bioengineered exosomes with curated cargo and membrane tags can be delivered intravenously (i.v.). Exosomes home to the site of injury and modify the targeted cell for repair. FB *Fibroblast*, EC *Endothelial cell*, CM *Cardiomyocyte*, M *Macrophage*, *SMC* Smooth muscle cell

The most effective therapeutic strategy for cardiac repair will likely incorporate specific loading and targeting of cardioprotective components into biologically-sourced exosomes^22,90,98^ or synthetically derived nanoparticles^99,100^. Despite a propensity for exosome uptake by monocytes/macrophages in vivo, multiple bioengineering approaches have made it feasible to target cells of interest. In the post-MI adult myocardium, it is foreseeable that a multipronged therapeutic approach targeting cardiomyocytes, macrophages, and endothelial cells with bioengineered exosomes will enhance the regenerative process (Fig. 4). To accomplish these goals, additional studies are needed to reveal the mechanism of cardiac macrophage-dependent regeneration, identification of therapeutic targets to enhance regeneration, and efficient methods of delivery.
